# Correlated activity supports efficient cortical processing

**DOI:** 10.3389/fncom.2014.00171

**Published:** 2015-01-06

**Authors:** Chou P. Hung, Ding Cui, Yueh-peng Chen, Chia-pei Lin, Matthew R. Levine

**Affiliations:** ^1^Department of Neuroscience, Georgetown UniversityWashington, D.C., USA; ^2^Institute of Neuroscience, National Yang-Ming UniversityTaipei, Taiwan

**Keywords:** object recognition, inferior temporal cortex, macaque, visual search, efficient coding

## Abstract

Visual recognition is a computational challenge that is thought to occur via efficient coding. An important concept is sparseness, a measure of coding efficiency. The prevailing view is that sparseness supports efficiency by minimizing redundancy and correlations in spiking populations. Yet, we recently reported that “choristers”, neurons that behave more similarly (have correlated stimulus preferences and spontaneous coincident spiking), carry more generalizable object information than uncorrelated neurons (“soloists”) in macaque inferior temporal (IT) cortex. The rarity of choristers (as low as 6% of IT neurons) indicates that they were likely missed in previous studies. Here, we report that correlation strength is distinct from sparseness (choristers are not simply broadly tuned neurons), that choristers are located in non-granular output layers, and that correlated activity predicts human visual search efficiency. These counterintuitive results suggest that a redundant correlational structure supports efficient processing and behavior.

## Introduction

Visual recognition engages neural mechanisms that are essential to our ability to learn and process complex information (Poggio and Bizzi, 2004). The key challenge of recognition is generalization, which requires that the representation is both object-specific and invariant to changes such as illumination and pose, even for novel objects. This is thought to occur via a hierarchy of cortical areas along the ventral visual pathway, ending in the inferior temporal (IT) cortex (Miyashita, 1993; Logothetis and Sheinberg, 1996; Tanaka, 1996; Tootell et al., 2003), but the underlying computations remain poorly understood (DiCarlo and Cox, 2007; DiCarlo et al., 2012). Current models and theories of recognition (Riesenhuber and Poggio, 1999; Masquelier and Thorpe, 2007; Mutch and Lowe, 2008; Bengio, 2009; Krizhevsky et al., 2012; Le et al., 2012; Zeiler and Fergus, 2014; Cadieu et al., 2014) are based on the idea that a hierarchy of simple and complex cells combine to increase specificity and invariance. To improve these models, it is necessary to understand the computations of local populations of neurons at an intermediate level of abstraction (DiCarlo et al., 2012).

A key concept is sparseness, a measure of coding efficiency. The current thinking is that sparseness increases efficiency by minimizing redundancy, correlation, and noise (Gawne and Richmond, 1993; Zohary et al., 1994; Vinje and Gallant, 2000; Olshausen and Field, 2004; Ecker et al., 2010; Renart et al., 2010; Xing et al., 2011; Hansen et al., 2012; King et al., 2013). Yet, reports in V1 slices and *in vivo* have shown the existence of neural ensembles that fire reliably in concert during spontaneous activity (Sadovsky and Maclean, 2014), and the same ensembles are active both without stimulation and in response to stimulation (Chu et al., 2014; Miller et al., 2014). We recently reported (Lin et al., 2014) that in macaque IT, correlated neurons “choristers” (Kenet et al., 2005; Carandini, 2014), neurons that have similar stimulus tuning and coincident spike timing, even during spontaneous activity, carry more generalizable object information than uncorrelated neurons (“soloists”). This surprising result hints that, counterintuitively, correlation *supports* efficient coding and that current thinking focused on sparsening, decorrelation, and denoising may be flawed.

The idea that the correlational structure, i.e., the spatial pattern of homogeneity vs. heterogeneity within a local population of neurons, may support efficient coding has been postulated in theory (Abbott and Dayan, 1999; Sompolinsky et al., 2001; Wu et al., 2002; Dehaene and Changeux, 2005; Averbeck et al., 2006; Cohen and Kohn, 2011; Ecker et al., 2011; Eyherabide and Samengo, 2013; Shamir, 2014), but it has received little experimental support. Three novel aspects of our study allowed us to explore this hypothesis.

**Figure 1:**
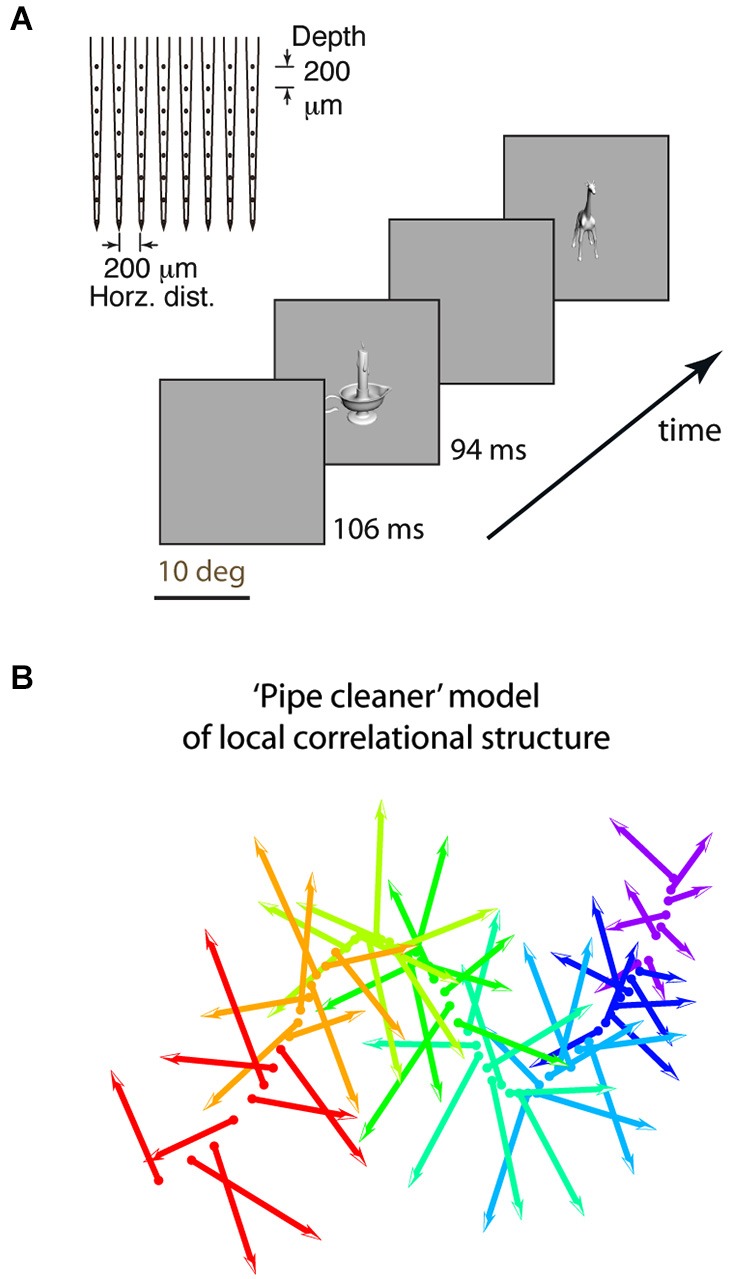
**Experimental design and “pipe cleaner” model. (A)** We inserted a dense multi-depth array (64 sites across ~2 cortical columns) in macaque lateral IT (A16) and recorded spiking responses under light neurolept anesthesia. Stimuli were presented via rapid serial visual presentation, for 94 ms ON and 106 ms OFF (5 Hz), in pseudorandom order for 10 repetitions. Spike count from 100 to 200 ms post stimulus onset was averaged across repetitions. **(B)** A “pipe cleaner” model linking local correlational structure in neighboring columns to invariant representation. Most neurons are weakly correlated “soloists” (the bristles), tied to an underlying structure of correlated neurons (“choristers”, the spine). Sampling a few points along the spine (a few choristers) is sufficient to reconstruct the overall structure. The model predicts that generalizable object information is carried by the choristers, and that the heterogeneity of the soloists may help to fine-tune the choristers to support generalization.

First, we used dense electrode arrays (64 sites across roughly two cortical columns, 0.2 mm resolution horizontally and in depth, Figure 1A) to characterize the correlational structure. High-density arrays allowed us to record neurons that have similar tuning, to measure redundancy as “Average Correlation Strength” (a site’s average pairwise tuning similarity with all other sites in the array, where the tuning similarity between two sites is the Pearson correlation of their z-normalized stimulus responses, related to the concept of “population sparseness” Willmore et al., 2011). Because previous reports of efficient coding had insufficient sampling density to measure population sparseness, they instead measured “sparseness” as tuning sharpness, the selectivity of a neuron’s response across stimuli, under the assumption that “sparseness” and “population sparseness” are interchangeable (that sparseness and correlation strength are inversely related) (Rolls and Tovee, 1995; Vinje and Gallant, 2000; Zoccolan et al., 2007; Willmore et al., 2011). When studies did examine functional correlation, it was in terms of individual pairs of neurons, without comparing the relationship between sparseness (tuning sharpness) and correlation (Gawne and Richmond, 1993; Sato et al., 2009; Takeuchi et al., 2011), or the comparison was limited to layers 2/3 (Tamura et al., 2014). Whether “correlation strength” and “sparseness” are related for a diverse sample of IT neurons remains untested (Willmore et al., 2011), and answering this question is important for understanding how local architecture relates to coding efficiency.

Second, the dense multi-depth arrays allowed us to examine layer specificity, which can tell us about input-output relationships. We and others (Sato et al., 2009; Lin et al., 2014; Tamura et al., 2014) previously reported that local IT populations have a correlational structure in which most neurons are weakly correlated and few neurons have strong tuning correlation and significant spontaneous coincident spiking (~6% of neuronal pairs in IT, vs. ~50% of pairs in V1; Chu et al., 2014). Yet, these rare IT choristers are also highly efficient. Just 4–5 choristers per array (the top 6% as defined by k-means clustering, or 8% as defined by average pairwise tuning correlation) have the same object coding capability, for within-category generalization, as the entire array population (no more are needed given their object coding efficiency; Figure 7C of Lin et al., 2014). Based on this correlational structure and the much-better object coding capability of choristers vs. soloists, we previously proposed a “pipe cleaner” model (Figure 1B, a “fiber bundle” in mathematical terminology) in which the choristers (the spine) are the substrate of IT’s output, encoding an invariant representation that supports generalization and recognition, and in which the soloists (the bristles) are IT’s inputs, acting as heterogeneous tensors that fine-tune this high-dimensional representation (in the parlance of DiCarlo et al. (2012), to support “cortically local subspace untangling” and to “flatten object manifolds”). If so, choristers and soloists should be layer specific, with soloists tending to be in input layers and choristers tending to be in output layers. Such layer-specificity would be consistent with reports of decorrelated responses near layer 4 of V1 (Ecker et al., 2010; Hansen et al., 2012) and with reports that tolerance but not selectivity (sparseness) increases along the ventral visual pathway (Rust and Dicarlo, 2010; Willmore et al., 2011).

Third, we tested whether local correlated activity can predict visual search efficiency for complex naturalistic object stimuli. Previous reports have linked IT neuronal tuning to visual perception (Logothetis and Schall, 1989; Op de Beeck et al., 2001; Baker et al., 2002; Sigala and Logothetis, 2002; Mruczek and Sheinberg, 2007a; Sripati and Olson, 2010; Verhoef et al., 2012) and have linked perception to topography in V1 (Michel et al., 2013), but the interpretation was not linked to correlational structure. If local correlational structure, e.g., from short-range lateral inhibition in IT, predicts search efficiency, it would support that correlated activity and topography are linked to complex shape perception. It would also support recent reports that abnormal correlated activity and excitatory/inhibitory balance in object areas are linked to abnormal perception in autistics, linking these findings to spiking activity (Jiang et al., 2013; Robertson et al., 2013). Here, we asked whether local correlated activity predicts visual search for combinations of naturalistic objects. To avoid effects that might be driven by spatial attention or processes earlier in visual cortex, we used brief presentations at random locations followed by masking, and we equalized the stimuli for low level visual properties such as Fourier energy. Also, our stimuli were object combinations defined by local correlated activity in IT (“neurally defined features”; each “feature” is a set of objects), rather than abstract human-defined shapes as in previous reports, so that the predictions are specifically tied to contrastive coding of complex features by neighboring IT columns (e.g., from lateral inhibition).

Together with our previous report (Lin et al., 2014), these tests provide additional support for the hypothesis that correlated activity supports efficient processing and behavior. We report that although the concepts of sparseness and decorrelation are often conflated, correlation strength and sparseness (when measured as tuning sharpness) should be considered as separate factors. We also provide additional support for choristers as the output neurons of IT, based on their cortical depth. Finally, we show that correlated activity in macaque IT predicts human visual search performance in a task with complex shapes.

## Methods

### Neurophysiology and stimulus presentation

All experimental procedures in monkeys (*Macaca cyclopis*) were performed in accordance with the National Institutes of Health Guide for the Care and Use of Laboratory Animals and were approved by the Institutional Animal Care and Use Committee of National Yang-Ming University. The procedures for the experiments were described in Lin et al. (2014) and are briefly summarized here. We inserted dense microelectrode arrays that had 64 sites (8 shanks and 8 contacts per shank, at 0.2 mm spacing spanning 1.4 × 1.4 mm horizontally and in depth, NeuroNexus A8×8-5mm-200-200-413) spanning all cortical depths and ~2–4 neighboring cortical columns (Figure 5 of Lin et al. (2014)). Recordings were made from 5 arrays, where each array was a separate insertion in a separate recording session, across 4 monkeys.

Initial surgery for headpost, EEG, and recording chamber implant was under isoflurane anesthesia, followed by repeated recording sessions under light neurolept anesthesia (Fujita et al., 1992; Wang et al., 2000; Tsunoda et al., 2001; Yamane et al., 2006; Sato et al., 2009, 2013; Brown et al., 2011) (0.9 μg/kg/hr i.v. Fentanyl, 70%/30% N_2_O/O_2_, 0.25 mg/kg i.m. droperidol, and 0.3–0.5% isoflurane) and muscle relaxation (1.2 mg/kg/hr i.v. rocuronium bromide). The fentanyl concentration is 100× lower than in a recent report that contrasted awake vs. anesthetized signals (Ecker et al., 2014), and 10× lower than in reports that did not find an effect on neuronal dynamics (Loughnan et al., 1987; Constantinople and Bruno, 2011). Our signals also lacked artifacts such as prolonged responses and up/down fluctuations reported with other anesthetics (Contreras et al., 1997; Haider et al., 2013). Compared to awake recordings, light anesthesia and muscle relaxation have the advantage of excluding potential effects from task-related top-down signals (Sigala and Logothetis, 2002; Maier et al., 2007; Ruff and Cohen, 2014) or eye movements (Rajkai et al., 2008; Ito et al., 2011), and a recent report suggests that activity during running resembles activity under anesthesia and is dissimilar to “visually detached” activity during quiet wakefulness (Froudarakis et al., 2014).

Single units were analyzed for coincident spiking and to remove cases of multiple detection of the same neuron across different contacts. All “site” responses were based on multi-unit activity (MUA) pooled from isolated single units at the same contact. We report only “site” responses because lower spike counts and the possibility of oversorting with single unit activity (SUA) can artificially weaken correlation measurements (Cohen and Kohn, 2011), and because the conclusions were the same as for MUA. The stimuli were 240 grayscale rendered objects or 113 colored photographed objects presented via rapid serial visual presentation (94 ms ON/ 106 ms OFF).

### Analyses

Analyses were based on spike count from 100 to 200 ms after stimulus onset. Each site’s tuning function was calculated as its trial-averaged response, z-normalized across stimuli. The same matrix of trial-averaged and z-normalized tuning responses across the array (i.e., a 240 × 64 matrix for 240 stimuli and 64 sites) was used as the input for correlation analysis, k-means, principal component analysis (PCA), and classifier analysis as described in Lin et al. (2014).

**Figure 2:**
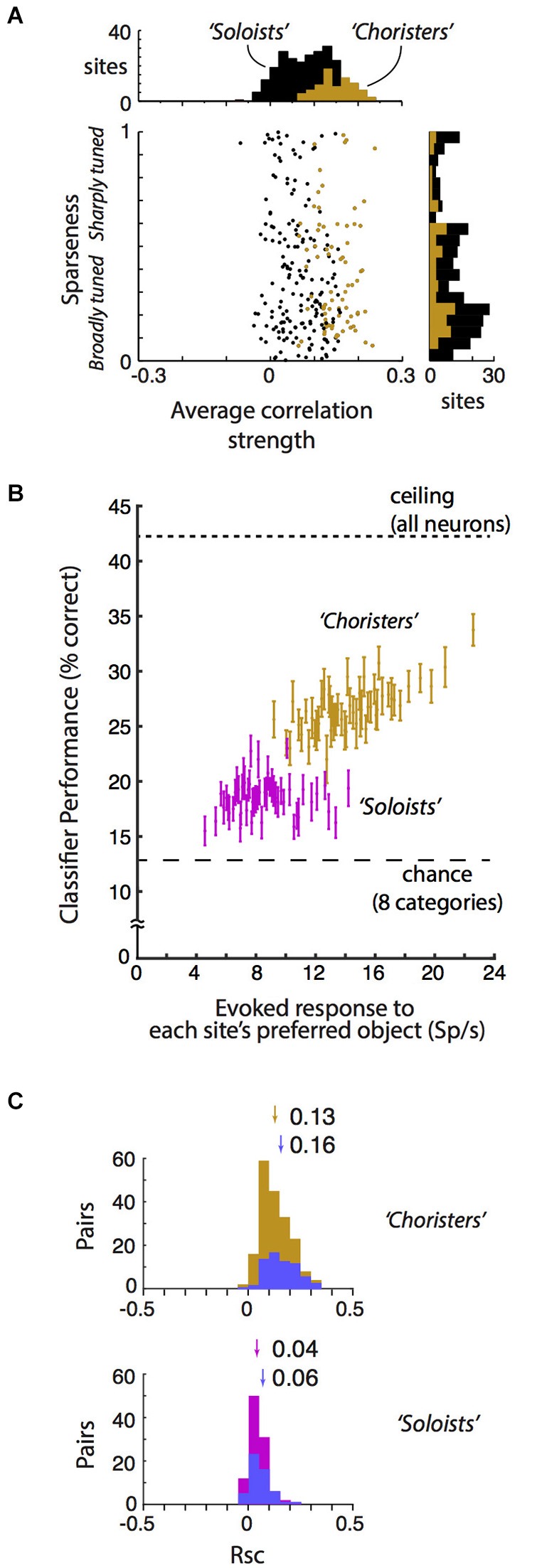
**Local correlational structure, sparseness, and generalization performance. (A)** Sparseness (tuning width) and average correlation strength were only weakly related across 250 sites in anesthetized IT (*r* = −0.09, *p* = 0.04). Sparseness was calculated according to Zoccolan et al. (2007) and Vinje and Gallant (2000). Average correlation strength was the average site-to-site tuning correlation between each site and all other sites in the same array. Sparseness and average correlation strength were each highly consistent across two stimulus sets (*r* = 0.72 and 0.70, *p* < 10^−37^ and *p* < 10^−39^ resp.). Choristers (brown) are the 30%ile of sites with the highest average correlation strength per array, and soloists (black) are the remaining sites. **(B)** Visual responsiveness vs. within-category generalization performance for choristers (top 30%ile, brown) vs. soloists (45–65%ile, red), for 2 sites per array, with at least 600 μm horizontal distance between sites. Visual responsiveness was calculated as the evoked (baseline-subtracted) response to each site’s preferred object, shown as the median across 10 sites (2 sites per array, 5 array insertions across 4 monkeys). Chance is 12.5% for 8 categories, and ceiling performance is based on all sites. Choristers and soloists were defined without test stimuli. Compare with Figure 7C of Lin et al. (2014). **(C)** Noise correlation (Rsc) of choristers vs. soloists (same colors and definitions as in **(B)** also with at least 600 μm horizontal distance). To control for visual drive, we also show Rsc for pairs of sites that have mean evoked response to each site’s preferred object between 10 and 30 spikes/s (blue). Arrows and numbers indicate mean Rsc.

**Figure 3:**
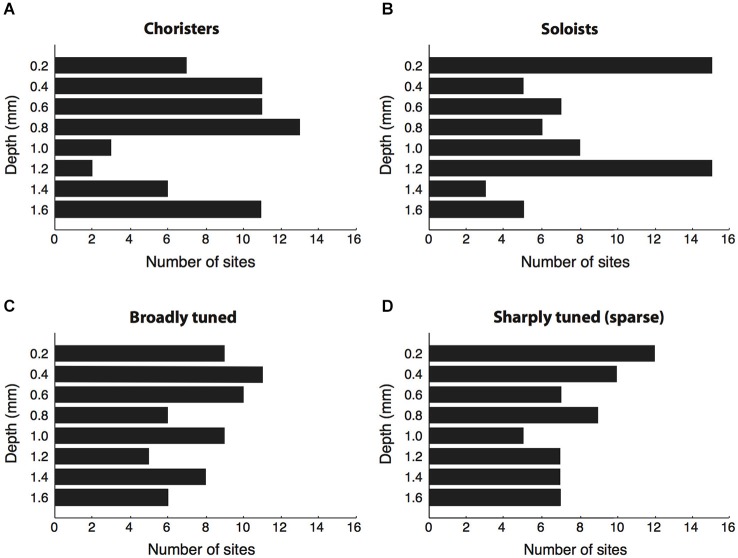
**Cortical depth vs. correlation strength and sparseness. (A,B)** We sorted sites by their average strength of tuning correlation with other sites in the same array, then grouped the top ~30% of sites per array as “choristers” and the bottom ~30% as “soloists”. Choristers are rarer in layer 4 (1.0–1.2 mm depth), whereas soloists are more common at 0.2 and 1.2 mm depth. The number of sites selected per group was higher for arrays 1–3 (16 choristers and 16 soloists per array) than for arrays 4 and 5 (8 choristers/soloists per array), because arrays 4 and 5 had fewer active channels. Average correlation strengths of choristers and soloists were 0.15 ± 0.04 and 0.02 ± 0.03, resp. **(C,D)** Same analysis based on sparseness (tuning sharpness). Average sparseness of broadly tuned and sharply tuned (sparse) sites were 0.12 ± 0.08 and 0.69 ± 0.25, resp.

We classified each site as a “chorister” or “soloist” based on the site’s average pairwise tuning correlation with other sites from the same array (the same calculation as in Lin et al. (2014) Figure 7C, brown line, but here “choristers” are random sites in the top 30%ile instead of rank-ordered sites in the top 8%ile). This top 30%ile corresponds to 16 sites per array for arrays 1–3 and 8 sites per array for arrays 4 and 5 that had more inactive sites. “Soloists” are the remaining sites (Figure 2A black dots). Choristers and soloists lie along a continuum of average pairwise tuning correlation strengths (Figure 2A). For object classification (Figure 2B) and noise covariation analyses (Figure 2C), we compared choristers (top 30%ile) against soloists in the 45–65%ile. For cortical depth (Figure 3B), the soloists are the bottom 30%ile. Layer-specificity is not seen for soloists in the 45–65%ile.

We used a linear support vector machine classifier to estimate the ability of a hypothetical downstream neuron (e.g., in prefrontal cortex) to read out the category of an untrained object (within-category generalization) based on the population activity in IT. The classifier output is based on the weighted sum of spiking activity from a set of IT neurons followed by a decision threshold. Because there were 8 possible categories, the classifier learned a one-vs.-all decision hyperplane for each of 8 categories and output the category that had the highest certainty.

Sparseness was calculated according to Vinje and Gallant (2000) and Zoccolan et al. (2007) as:

$$S=\left(1-\frac{{\left(\sum \frac{R_{i}}{n}\right)}^{2}}{\sum \frac{R_{i}^{2}}{n}}\right)/\;\left(1-\frac{1}{n}\right),$$

where R*_i_* is the site response to the i-th stimulus and n is the number of stimuli in the set.

We could estimate the cortical depth because we were able to visually see individual sites disappear into the brain during insertion and, because of the small footprint of the array shanks (15 μm thick, 33 μm wide), we could also track individual units as they transitioned from the deepest to the most superficial sites during array insertion. Anatomical confirmation of depth was impossible due to damage from later recording sessions. However, we estimate that the deviation of the array from vertical was less than 8 deg (less than 0.2 mm horizontal offset at the deepest site), based on anatomical confirmation of our V1 recordings using the same arrays (Supplemental Figure 1 in Chu et al., 2014).

For PCA, each PC consists of relative site activities (e.g., 1 × 64 matrix of coefficients for 64 sites, normalized to unit length) and stimulus-related scores (e.g., 1 × 240 matrix of weights for 240 stimuli) for that PC. The z-normalized response of a site to a stimulus can be back-calculated by summing, across all PCs, the product of the site’s coefficient for each PC and the stimulus’s score for that PC.

### Human testing

#### Observers

Procedures were approved by the Institutional Review Board of Georgetown University and informed consent was obtained from all observers. Six observers (3 male, 3 female, including the second author) participated in the experiments. All observers had normal or corrected-to-normal vision. Apart from the second author, observers were naïve as to the purpose of the experiment and were paid for participation.

#### Apparatus

Stimuli were controlled by computer using Matlab and Psychtoolbox 3 (Kleiner et al., 2007) and displayed on a 17″ cathode ray tube (CRT) (Sony Trinitron Multiscan 17sfII) with spatial resolution 1024 × 768 pixels and refresh rate of 60 Hz. Eye-screen distance was 57 cm, so that each pixel subtended approximately 0.03°. Ambient illumination was <4 Cd/m^2^.

#### Stimuli

Object stimuli belonging to neurally defined features (grayscale rendered objects, “Set 1”) were resized to 64 × 64 pixels (1.9° × 1.9°) and convolved with a 3 × 3 pixel Difference-of-Gaussians filter to match the background gray. Because the IT correlational structure is slightly more stable across stimulus sets for z-normalized responses than for raw responses, we constructed stimuli using the neurally defined features from z-normalized responses. Object stimuli were then equated for low-level image properties using the SHINE toolbox (Willenbockel et al., 2010). Groups of object stimuli were then randomly tiled to create the background (5 different objects), target (3 different objects), and distractors (3 different objects). Tiling position combinations were restricted to avoid lines of the same object. Target and distractors luminances were darkened by 5%, to make them more visible against the background. Mask stimuli were specific for each trial, created by scrambling the background (without target and distractors) at 0.24° resolution. Fixation point was a black square of 0.45° × 0.45°.

#### General procedure

Each block consisted of 144 trials comprising 48 “opposite”, 48 “related”, and 48 “unrelated” conditions, all with the same stimulus onset asynchrony (SOA) and adaptation duration. To minimize effects spanning across trials, each trial was preceded by an inter-trial interval (trial was initiated by key press), a blank fixation screen (1.5 s), and an adapting background of up to 8 s. In addition, objects were balanced across targets, distractors, and background and across conditions.

## Results

The neurophysiological data here is based on reanalysis of a previously reported dataset collected in monkeys under light neurolept anesthesia (Lin et al., 2014). Briefly, analyses are based on trial-averaged z-normalized responses (250 multi-unit “sites” and 6462 site pairs from 359 neurons) to stimuli that were presented via rapid serial visual presentation (Figure 1A). We begin by addressing a few concerns about our previous report: that the 6% cutoff of choristers is arbitrary and that in fact “choristers” and “soloists” are not two types of neurons, and that perhaps the better object coding performance of choristers is due to multiple detection of the same neuron across contacts, or because soloists are less visually driven. In fact, the distribution of average correlation strengths is continuous, and the separation into “choristers” and “soloists” is merely for convenience of comparison, not to say that there are two distinct cell types. Correlation strength and within-category generalization performance both decline smoothly, so missing a few top “choristers” during sampling should not affect the resulting structure very much. To increase the population size for testing the effect of average correlation strength, we relaxed the definition of choristers as random sites in the top 30%ile of average pairwise correlation strength per array (Figure 2A, brown), and of soloists as random sites in the median 30%ile (45–65%ile; black is lower 70%ile). This 30%ile threshold for choristers corresponds to minimum average correlation strengths of 0.12, 0.15, 0.16, 0.09, and 0.06 for the 5 arrays. These thresholds are similar for sites separated by at least 0.6 mm horizontal distance (0.09, 0.11, 0.14, 0.07, and 0.05). Although correlated neurons do tend to be more visually driven than uncorrelated neurons (c.f. Figure 5C of Tamura et al. (2014)), we still observed higher performance for choristers when choristers and soloists were matched for visual drive (Figure 2B, ~12 Sp/s baseline-subtracted response to each site’s preferred stimulus; based on 5 arrays and 2 sites per array, at least 0.6 mm horizontal distance between sites).

### Sparseness and correlation strength are mostly unrelated

In previous reports, sparseness (measured as tuning sharpness) was thought to support efficient coding by reducing correlated activity (Young and Yamane, 1992; Rolls and Tovee, 1995; Baddeley, 1996; Olshausen and Field, 1996; Bell and Sejnowski, 1997; Vinje and Gallant, 2000; Zoccolan et al., 2007). This would predict that soloists should have better object coding capability (whereas our results suggest that choristers have better object coding, at least for within-category generalization) and that soloists should be sharply tuned. Conversely, a trivial explanation of the better object coding capability of choristers is that perhaps choristers are broadly tuned and therefore have better tolerance to stimulus variations.

We report that neither prediction is correct. Sparseness (measured as the modified sparseness index of Vinje and Gallant (2000), Lin et al. (2014) and average correlation strength are mostly uncorrelated across sites. Within each of 5 arrays (5 separate array insertions across 4 monkeys), the relationship between sparseness and average correlation strength was non-significant, and it was weak and barely significant when pooled across all arrays (Pearson *r* = −0.09, *p* = 0.04, *N* = 250 sites, Figure 2A). This weakness was not due to noise in either measurement, because sparseness and average correlation strength were each highly consistent across two stimulus sets (*r* = 0.72 and 0.70, *p* < 10^−37^ and *p* < 10^−39^, resp.). This dissociation between sparseness and correlation is consistent with a previous conjecture that these measures are unrelated (Willmore et al., 2011) and with a recent report that found a weak (albeit positive, *r* = 0.07, *p* < 0.001, rather than negative) dissociation in layer 2/3 (Tamura et al., 2014). Our data show that the dissociation also holds for a wider sample of IT neurons across supragranular, granular, and infragranular depths.

### Correlated neurons are mostly in output layers

A key issue in linking neural activity to models is the cortical layer of different functional elements. An ongoing debate is whether neurons are correlated or uncorrelated, and whether these are in input or output layers. In V1, a recent study suggested that noise correlations are much lower than previously thought (Ecker et al., 2010), but alternatively it has been reported that noise correlation is layer-dependent and is lower, with better coding efficiency, in the granular layer (Hansen et al., 2012).

We suggest that neither view is entirely correct in IT. Here, we report that correlated neurons (choristers, with more efficient coding) are almost exclusively found in supragranular and infragranular layers. In IT, signal (tuning) correlation and noise correlation are related, and choristers tend to have stronger noise correlation (choristers (brown): Rsc = 0.13; soloists (45–65%ile, red): Rsc = 0.04; *p* < 10^−22^, unpaired *t*-test; Figure 2C), including pairs separated by at least 0.6 mm horizontal distance and with similar visual drive (mean baseline-subtracted response to preferred stimulus of each cell is between 10 and 30 spikes/sec) (choristers: Rsc = 0.16; soloists: Rsc = 0.06; *p* < 10^−13^; blue).

Of the 64 choristers, most were in supragranular and infragranular layers and only five were between 1.0–1.2 mm depth, near layer 4 (Figure 3A). Conversely, the most uncorrelated soloists (the ~30% of sites with the lowest correlation strength per array) were more prevalent at 0.2 and 1.2 mm depth (layers 1 and 4), although roughly half were in supragranular and infragranular layers (Figure 3B). The result was similar for single-unit activity. The proportion of choristers vs. soloists was significantly lower in the granular layer (1.0–1.2 mm) compared to supragranular and infragranular layers (*p* = 0.0007 and *p* = 0.0003, two-sided Fisher’s test), and the difference between supragranular and infragranular layers was non-significant. This layer-specificity is consistent with a recent report in V1 that also measured correlated variability (Hansen et al., 2012). In contrast to correlation strength, sparseness, a measure of coding efficiency that is commonly based on tuning sharpness (Young and Yamane, 1992; Rolls and Tovee, 1995; Vinje and Gallant, 2000; Zoccolan et al., 2007), was not layer specific (Figures 3C,D, n.s. for all comparisons).

### The low dimensional correlational structure is also in output layers

A recent perspective article (DiCarlo et al., 2012) highlighted the need to understand the processing of local populations of neurons at an intermediate level of abstraction. Covariation analysis (e.g., k-means clustering and PCA) is a useful form of abstraction because it directly ties the correlational structure to the idea of a low-dimensional manifold representation of object features (DiCarlo et al., 2012) and to our pipe cleaner model (Lin et al., 2014). We often encounter novel objects and novel environments (Vaziri et al., 2014) that must be categorized, and it is thought that the visual system learns useful shape statistics of the animal’s environment (Srihasam et al., 2014). A key concept of the model is that the invariant representation, which supports generalization across rotation-in-depth, changes in illumination, and variations within an object category (studied here), has a spatial organization that is concentrated in a low-dimensional correlational structure. Such a low-dimensional correlational structure could be very useful for decoding by downstream neurons and for generalization learning, by providing a smoothly differentiable structure that is stable across categories, by reducing the number of inputs that must be pooled (instead of listening to all neurons, a downstream neuron could conceivably identify the most useful neurons in a population based solely on coincident timing, even during spontaneous activity), and by supporting robustness for noisy spiking populations.

We previously used k-means clustering (Lin et al., 2014) to identify clusters of sites that behaved more similarly across stimuli. Note that this is different from the typical approach, where the same data is clustered as groups of stimuli according to their response similarity (Kiani et al., 2007). Also, to focus on the local correlational structure, we focused our analysis specifically within each array, rather than pooling across the entire population (all arrays). Here, we extend our approach to PCA, to tie the low-dimensional structure to output layers and to behavior. Because many of the conclusions drawn from PCA regarding spatial structure are similar to those from k-means clustering and from pairwise site correlations, we will discuss only the highlights below.

Compared to k-means clustering, PCA has the advantage of explaining more of the variance using fewer dimensions, because each PC is exactly aligned to maximally explain the remaining variance, whereas k-means clustering will include sites that are uncorrelated (soloists). Thus, whereas k-means clustering tends to highlight columnar organization (changes in tuning across cortex), PCA can reveal the layer-specificity of the low-dimensional correlational structure. For these dense arrays, the spatial patterns of site covariation were nearly identical between the lower PCs and k-means clustering and corresponded roughly to the differential activation of neighboring columns (at higher PCs, it is less likely that the orthogonal PCs are relatable to biological processes). Specifically, PC1 (the dimension of maximum variance within an array) corresponds roughly to activation vs. suppression of most sites in the array, which may contribute to invariant representation by encoding how strongly a feature or feature contrast is present within an object category. PC2 (the dimension that best explains the remaining variance) corresponds roughly to the differential activation of two neighboring cortical columns (i.e., the sign and relative strength of a feature contrast) and appears virtually identical to k-means clustering at *k* = 2 (Figure 5 of Lin et al., 2014).

**Figure 4:**
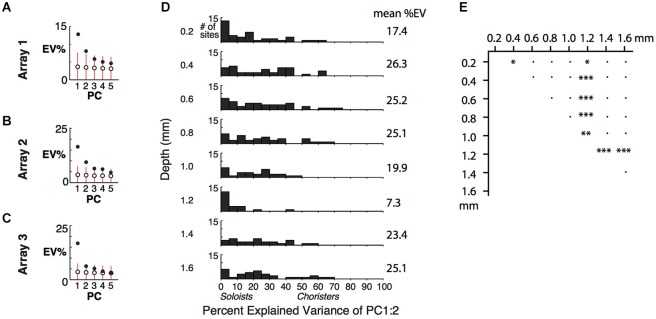
**Cortical depth vs. low-dimensional correlational structure. (A)** Percent of response variance explained by PCs 1–5, based on z-normalized responses. Chance and 5–95%ile distributions are indicated by open circles and red bars, based on shuffling of response IDs across trials. **(B,C)** Explained variance for Arrays 2 and 3, from two separate array insertions (separate recording sessions) in monkey 2. **(D)** Cortical depth vs. percent explained variance of PCs 1 and 2 across 5 arrays. **(E)** Comparison of distributions in **(D)** among different depths. *p < 0.05, **p < 0.01, ***p < 0.001, ^●^*p* = n.s.

By examining how well the lowest PCs explain the variance of individual sites, we can determine the layer-specificity of the low-dimensional correlational structure. Because of the high proportion of soloists and the rarity of choristers (Figure 2A and Gawne and Richmond, 1993; Sato et al., 2009; Lin et al., 2014), the first few PCs explained only a small fraction of the total variance within each array (Figures 4A–C, example arrays 1–3; responses were averaged across repetitions and z-normalized), even though the tuning of individual sites was highly consistent across even vs. odd trials (*r* ~ 0.6–0.9, Figure 2A of Lin et al. (2014)). The first two PCs explained only about 25% of the response variance across stimuli. A previous study reported 15% explained variance for two PCs, based on recordings from random penetrations across IT (Baldassi et al., 2013), and another study reported ~70% for two PCs (~60% for PC1) based on recordings from electrode bundles targeted to the centers of IT optical imaging domains (Figures 10, 16 of Sato et al. (2009)). A possible explanation for the large difference across studies is that in addition to layer-specific heterogeneity of choristers vs. soloists, there is also topographical heterogeneity that cannot be attributed to bias from image guided electrode targeting.

For individual sites, the percent of variance explained (%EV) by the first two PCs varied widely and was lower for sites in layers 1 and 4 (0.2 and 1.2 mm depths) (Figures 4D,E). It was more widely distributed, with up to 77%EV, for sites in supragranular and infragranular layers. The specificity of the correlational structure to output layers hints that it is shaped by local networks, rather than by feedforward or thalamic input to layer 4 or feedback from higher areas to layer 1. The lower explained variance at 0.2 mm and 1.2 mm depths (many sites are <5%EV), despite good even-vs.-odd trial tuning consistency at all depths (Figure 2A of Lin et al. (2014)), suggests that the inputs to layers 1 and 4 are nearly orthogonal to the correlated activity, consistent with our “pipe cleaner” model. In comparison, scrambling the stimulus IDs of the scores of the first two PCs, without altering the PCA coefficients, resulted in −23%EV on average (i.e., the difference between the scrambled prediction and the actual response has a total variance that is on average 123% that of each site’s actual total variance, Figure 4D). The fact that the EVs of scrambled predictors are negative, and not zero, for sites in all layers further supports that these sites are visually driven and selective. Only layer 4 (1.2 mm depth) had average response below baseline (−0.5 spikes/s), consistent with suppression by local or feedforward inhibition. Overall, this result extends upon the layer-specificity of choristers and soloists as measured by average correlation strength by showing that the correlational structure is concentrated in a few dimensions (a low-dimensional manifold), mainly in a subpopulation of neurons in output layers.

### Neurally defined features based on correlated activity

To link the correlational structure to behavior, we constructed “neurally defined features” based on the tuning of neighboring IT columns. Previous reports used a variety of methods (feature reduction, k-means clustering, PCA, or simply averaging the tuning along a penetration) to characterize IT tuning (Young and Yamane, 1992; Tsunoda et al., 2001; Baldassi et al., 2013; Sato et al., 2013). However, because their analyses focused on the tuning of single neurons or random IT populations, behavior has not been tied to the concept of a cortically local low-dimensional manifold and lateral inhibition.

Here, to focus on the differential coding by local populations, we defined “neurally defined features” as sets of stimuli determined by PCA of each array (the same PCs as in Figure 4, e.g., computed from a 240 stimuli × 64 site matrix of z-normalized tuning responses). The “neurally defined features” are sets of stimuli with extreme PCA scores, treated collectively without altering or blending the images. For example, feature “Array 1 PC1+” is the set of 10 stimuli with the most positive PC1 scores for Array 1, allowing that some of the same stimuli may also belong to PC2+ or PC2− or to features of another array (however, for behavioral testing we did not allow “reuse” of stimuli across background, target, and distractor within a trial). Although one “Array 1 PC1+” stimulus may have a higher PC1 score than another “Array 1 PC1+” stimulus, we treat them equally because it is the collective effect of the set of “Array 1 PC1+” stimuli that dilutes away stimulus-specific effects, to increase the feature’s specificity to that neuronal population (e.g., vs. populations in early visual cortex or elsewhere in IT). In each array, PC2+ and PC2− correspond to differential activation of neighboring cortical columns, and PC1+ and PC1− correspond to co-activation and co-suppression of most neurons in the array (differential activation at a larger spatial scale).

**Figure 5:**
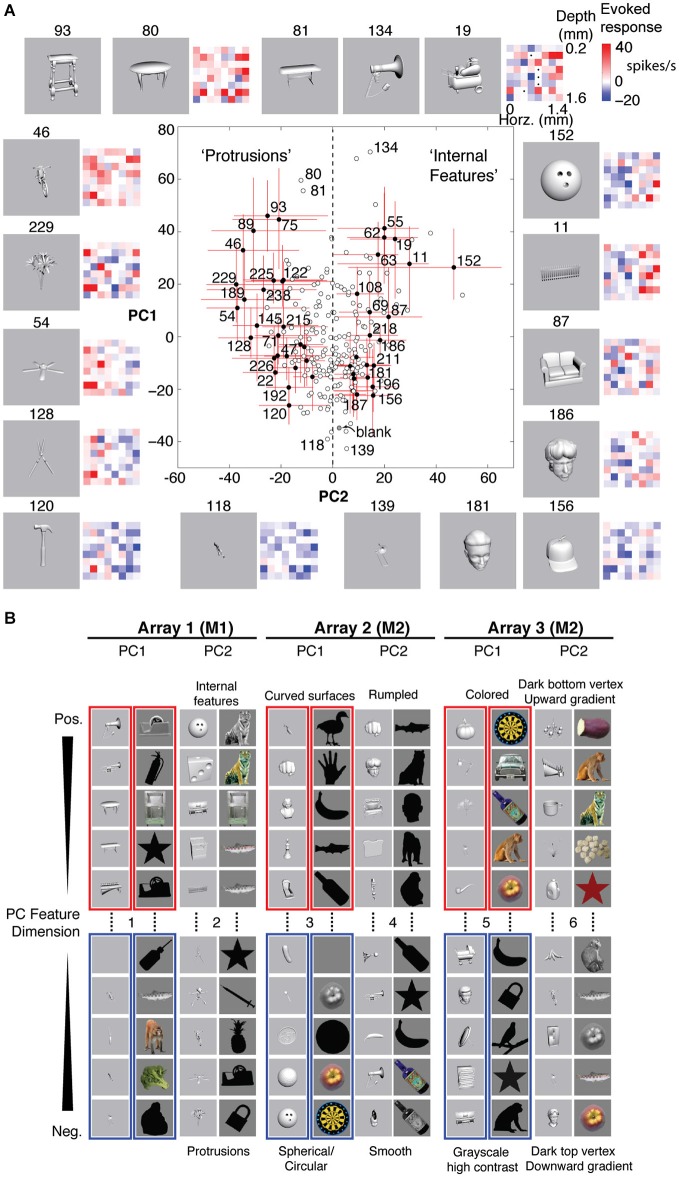
**Neurally defined features. (A)** Neurally defined features based on Array 1’s PC1 and PC2 scores. Each red and blue 8 × 8 matrix shows baseline-subtracted response to one stimulus across the 64 sites, spanning all depths and neighboring IT columns. PC1+ stimuli activated most sites. PC2+ and PC2− stimuli differentially activated sites on the right and left sides of the array. Numbers indicate stimulus IDs. Black dots in stimulus 19’s matrix indicate inactive sites. Red lines indicate 5–95%ile, and filled circles indicate stimuli with significant PC2. “Protrusions” and “Internal Features” are labels to help see the pattern of PC2− and PC2+ stimuli, but the labels are not part of the feature definition. **(B)** Features from 3 array insertions (3 recording sessions) in two monkeys and two stimulus sets. Only the stimuli with the most extreme scores are shown, out of 240 object stimuli for set 1 (grayscale rendered 3D objects) and 113 stimuli for set 2 (color, grayscale, and silhouette photographs). The slight difference between panels **(A)** and **(B)** is because the scores in A are calculated from unnormalized responses, to scale with the baseline-subtracted firing rates in the matrices, whereas the scores in **(B)** are from z-normalized responses, for better consistency of spatial covariation patterns across stimulus sets.

Figure 5A shows examples of stimulus responses along PCs 1 and 2 for Array 1. The red and blue matrices show examples of baseline-subtracted firing rates across the 8 × 8 array to specific stimuli. Across different levels of overall activation and suppression (different PC1 scores), stimuli that differentially activated the left column more than the right column (PC2− stimuli) tended to be objects with protrusions, whereas stimuli that differentially activated the right column more than the left column (PC2+ stimuli) tended to be objects with internal features. Although these semantic descriptions are qualitative and are not part of the feature definition, the positive and negative PC extremes appeared to prefer contrastive features (“rumpled” vs. “smooth”, “upward” vs. “downward vertex with gradient”) that were consistent across two stimulus sets (grayscale rendered objects and color/grayscale/silhouette photographed objects). Therefore, we assigned the positive and negative extremes to separate features, resulting in 12 neurally defined features derived from 6 PC feature dimensions (PCs 1 and 2 from 3 arrays, i.e., 3 separate recording sessions across 2 monkeys, Figure 5B).

Why use PCA, instead of k-means clustering or penetration averaging? Because neighboring columns have correlated tuning and because k-means clustering does not distinguish soloists from choristers, features from one k-means cluster are less visually distinguishable from those of neighboring clusters. Unlike PCA, k-means clustering or penetration averaging would have ordered stimuli according to how strongly they activated each column, causing stimuli that appear very different to group together (e.g., the bike (#46) and the fence (#11) for the right column, or the shears (#128) and the couch (#87) for the left column). PCA features appear more different, particularly PC2+ vs. PC2−. We note that this advantage of PCA may be specific to local populations sampled by densely spaced electrode arrays. The differential coding along PC2 is consistent with previous reports of a “shape-contrast” effect in perception (Suzuki and Cavanagh, 1998) and in IT responses (Leopold et al., 2006), although it is distinct from the idea of norm-based encoding (Valentine, 1999) because it is primarily driven by shape rather than by semantic category or low-level properties such as color and texture (Baldassi et al., 2013; Lin et al., 2014).

These PC feature dimensions were uncorrelated across arrays (Pearson correlations of PC scores were non-significant), even the features measured from different sessions 3 mm apart in the same monkey (M2), indicating that the features are not simply due to familiarity (Mruczek and Sheinberg, 2005, 2007b; Hein et al., 2007; Anderson et al., 2008) or coarse topography (Op de Beeck et al., 2007; Sato et al., 2013). Also, the monkeys had never seen these stimuli previously.

### Neurally defined features predict visual search efficiency

**Figure 6:**
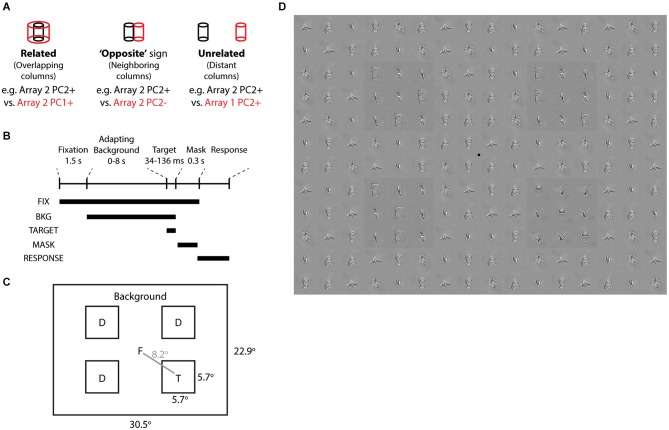
**Visual search task based on neurally defined features. (A)** “Related”, “Opposite”, and “Unrelated” conditions are tied to the differential activation of overlapping, neighboring, and distant IT columns by neurally defined features. In each condition, the target objects belong to one feature (e.g., Array 2 PC2+) and the distractor and background objects are disjoint sets belonging to the other feature (e.g., Array 2 PC2−). “Related” features are from different PCs of the same array. “Opposite” features are from opposite signs of the same PC of the same array. “Unrelated” features are from different arrays. **(B)** Time course of each trial. Following Fixation screen and Adapting Background (0–8 s), Target and Distractors appeared for 34–136 ms, followed by a Mask with tile-scrambled background images. After disappearance of the fixation point, subjects reported via keypress the target quadrant. **(C)** Each trial consisted of one target and 3 distractors at four possible locations. Objects and target locations were balanced across all conditions. **(D)** Example stimulus from “opposite” condition, with target in quadrant 4. Distractors and background are from Array 3 PC2−. Target is from Array 3 PC2+. All object stimuli were matched for low level image properties via the SHINE toolbox. To aid target localization, a luminance pedestal was added to target and distractors.

To link correlated activity to behavior, we designed a human visual search task in which the target, distractors, and background were disjoint sets of objects from monkey neurally defined features. Previous reports based on simple features such as orientation, color, and size hint that visual performance is associated with horizontal processes and lateral inhibition in early visual cortex (Butler et al., 2008; Yoon et al., 2010; Michel et al., 2013). Here, we asked whether lateral inhibition among complex feature representations in IT might also predict visual performance. Because of the short horizontal range of lateral inhibition in macaque IT, which may translate to longer-range inhibition in humans if similar statistical feature mechanisms are useful for representation, we tested whether the target would be more salient from the background if they were contrastive (“opposite” sign) features from the same array (differentially activating neighboring columns, e.g., Array 2 PC2+ target vs. PC2− background), than if they were “related” features (different PCs of the same array, activating the same column at different scales, e.g., Array 2 PC2+ target vs. PC1+ background) or “unrelated” features (PCs from different arrays, activating distant columns >3 mm apart, e.g., Array 2 PC2+ target vs. Array 1 PC2+ background) (Figure 6A). The distractors and background were disjoint sets of objects sharing the same neurally defined feature.

To induce a temporary visuoperceptual distortion as in Leopold et al. (2001), we adapted the subject to the background for up to 8 s, followed by a brief (34–136 ms) presentation of the target and distractors and then a mask (scrambled background) (Figures 6B,C). Subjects indicated via key press the quadrant in which the target appeared. Subjects were instructed to search for the quadrant whose pattern appeared different from the other three quadrants. We measured visual search efficiency as the reporting accuracy of the target quadrant (chance = 25% correct). Such visual search displays are commonly used to study early perceptual processes and have only recently been applied to neurally related complex shapes (Sripati and Olson, 2010). A strength of the task is that the brief stimulus appearance and the target location randomization preclude artifacts from differences in spatial attention or eye position. To focus the task on complex shapes rather than early visual processes, we used the SHINE toolbox (Willenbockel et al., 2010) to equalize the objects in terms of low-level cues including luminance, contrast, and orientation-specific Fourier power (including spatial frequency) (Figure 6D).

**Figure 7:**
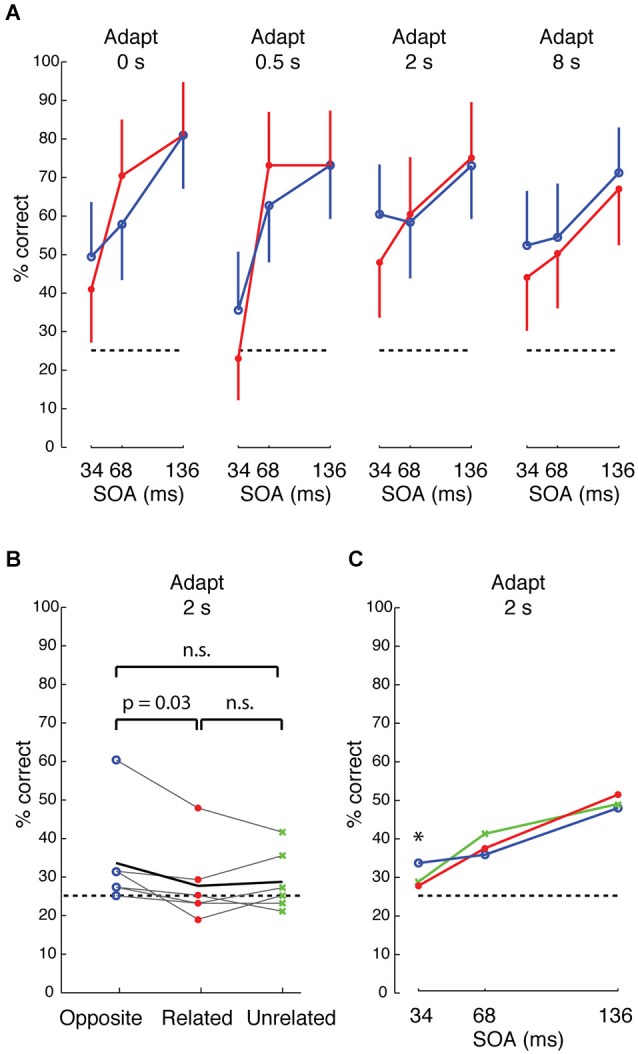
**Visual search performance for neurally defined features. (A)** Performance of one subject for target whose neurally defined feature is “opposite” (blue) or “related” (red) to that of the distractors and background, across different adaptations and different stimulus onset asynchrony (SOA) between stimulus and mask. Accuracy is higher for “opposite” at 34 ms SOA, and the difference between “opposite” and “related” is more consistent at longer adaptation. Error bars show 95% CI, based on 48 trials per condition. **(B)** Performance across 6 subjects at 2 s adaptation and 34 ms SOA for “opposite” (blue), “related” (red), and “unrelated” (green) conditions. Black line indicates average performance across subjects. The difference between “opposite” and “related” is significant at *p* = 0.03, based on Wilcoxon signed ranks test. **(C)** Average performance across 6 subjects at 34, 68, and 136 ms SOA, 2 s adaptation.

We began by comparing, in one subject, how performance depended on stimulus condition, target duration (stimulus onset asynchrony “SOA” between target/distractors and mask) and adaptation duration. At 34 ms SOA, performance was consistently higher across different durations of adaptation when the target feature was “opposite” in sign to the distractors and background (i.e., when target and background were contrastive features that differentially drive neighboring IT columns) (Figure 7A, blue) than when target and background were “related” features (red) (*p* = 0.038, Cochran-Mantel-Haenszel test). Although performance was slightly higher at 2 s adaptation, the odds ratios were not heterogeneous across different levels of adaptation (*p* = n.s., Breslow-Day test). An opposite effect was seen at 68 ms SOA at 0 and 0.5 s adaptation, but the effect reversed with longer adaptation. At the longest adaptation (8 s), performance was higher for “opposite” than for “related” features at all SOAs. Based on this pattern, we surmised that the effect was most reliably consistent with the prediction at the shortest SOA (34 ms) and with longer adaptation. To avoid tiring the subjects with the 8 s long adaptation or a possible flooring effect at shorter adaptation (e.g., 0.5 s), we tested all subjects at 2 s adaptation.

Across 6 subjects, visual search efficiency was consistently higher for “opposite” features than for “related” features at 34 ms SOA and 2 s adaptation (Figure 7B, *p* = 0.03, Wilcoxon signed rank test). As with the first subject, the higher performance for “opposite” features was only observed at the shortest SOA of 34 ms across the 6 subjects (Figure 7C). The persistence of the effect across different durations of adaptation at the short 34 ms SOA hints that the effect is likely driven by feed-forward processing and short-range lateral interactions in IT, because 34 ms is likely too brief for feedback (Bansal et al., 2014; Scholl et al., 2014) or long-range lateral interactions (Singer and Kreiman, 2014; Tang et al., 2014). We suggest that the mechanism is associated with short-range lateral inhibition (e.g., between neighboring columns) in IT, similar to reports of lateral interactions in early visual cortex (Das and Gilbert, 1999; Michel et al., 2013), rather than a distance-dependent effect in IT, because the search efficiency of “related” (0 mm cortical separation) and “unrelated” (>3 mm separation) features was not significantly different. Also, “opposite” features had higher search performance than “unrelated” features in 4 of 6 subjects, but this difference did not reach significance.

## Discussion

Our results suggest that correlated activity contributes to efficient coding and human visual search efficiency. The main findings are that correlation strength and sparseness are only weakly related and should be considered as separate factors, that correlated activity is primarily located in output layers, and that correlated activity in monkey IT predicts human visual search efficiency. Together, these results suggest that correlated activity may be the substrate of IT’s output and that, contrary to previous reports, correlated activity contributes to coding efficiency.

### “Population sparseness” vs. “sparseness” in efficient coding

These results suggest that a fundamental shift is needed in our approach to understanding efficient coding. Previous reports of efficient coding assumed that population sparseness and tuning sharpness (conventionally termed “sparseness”; Rolls and Tovee, 1995; Vinje and Gallant, 2000; Zoccolan et al., 2007; Willmore et al., 2011) are interchangeable. Instead, our results suggest that correlation strength (inversely related to population sparseness) is better than tuning sharpness as a measure of population redundancy, and that these two measures are mostly unrelated. Surprisingly, they show that the representation is more correlated in output layers than in input layers, which is opposite to the expectation that increasing sparseness supports efficient coding. This layer-specific increase in correlation is unrelated to tuning sharpness. Together with our previous report showing the better object coding capability of choristers vs. soloists, these results highlight the role of correlation in efficient coding.

### Why are correlation strength and sparseness unrelated?

The main reason for this apparent discrepancy is that previous studies did not measure population sparseness. Their wider electrode spacing meant that neuronal tuning was too dissimilar to compute population sparseness. Dense sampling, on the order of 64 neurons per mm^3^, is necessary to measure population sparseness, because neuronal tuning is heterogeneous even within a cortical column and because choristers are rare (Sato et al., 2009; Lin et al., 2014). It is unclear what mechanism might enable correlation of sharply tuned neurons in output layers and decorrelation of broadly tuned neurons in input layers. The prevalence of soloists in input layers 1 and 4 suggests that the feedforward and feedback inputs to IT are already decorrelated, or that they are actively decorrelated by inhibition. Conversely, our finding that correlated activity is mostly in output layers is consistent with the layer specificity of local circuits and horizontal fibers. However, the consistency of our V1 and IT results in terms of tuning and spike timing correlational structure suggest that they are probably driven more by local circuitry than by long range fibers, which have different patterns in V1 vs. IT (Tanigawa et al., 2005).

### Implications for visual search efficiency

Overall, these results support that a human homolog of IT, previously shown by many studies (Grill-Spector et al., 2001; Tootell et al., 2003; Orban et al., 2004; Kriegeskorte et al., 2008), guides search based on complex features. In relation to classical theories of visual search based on feature integration theory (FIT; Treisman and Gelade, 1980), these results differ in two key aspects. First, whereas FIT posits that fast visual search relies on early visual areas, our results support an accumulating body of evidence that later visual areas also contribute to fast visual search (Hochstein and Ahissar, 2002). Second, FIT posits that pre-attentive, parallel search is more efficient for low-level features than for feature conjunctions. Our results show that preattentive, parallel search is also more efficient for specific types of complex features, contextually dependent on the complex features present in the background, and that this contextual dependency is specifically linked to cortical neighborhood relationships and correlated activity in IT. This supports a model by Duncan and Humphreys that all search is parallel and depends on representational similarity and competition for resources across multiple levels of the visual system (Duncan and Humphreys, 1989, 1992).

Consistent with a previous report that linked macaque IT responses to human visual search efficiency (Sripati and Olson, 2010), our results suggest that this context dependency is due to a stimulus-specific competition for resources that can be explained by local contrastive mechanisms such as lateral inhibition (Wang et al., 2000; Leopold et al., 2001). Our results strengthen the case that this mechanism is tied to competition for local resources in IT, vs. in earlier areas, because it depends on IT cortical proximity. Also, by linking search efficiency to correlational structure, our results support an assumption in the previous report (Sripati and Olson, 2010), that a population of heterogeneous neurons (e.g., within an IT column) can be modeled by the discriminative capacity of their correlated activity, as the activity of a few neurons (choristers). One difference from the previous report (Sripati and Olson, 2010) is that their behavioral and neural responses were predicted by the coarse footprint difference of the objects, i.e., the spatial overlap of the blurred images, whereas in our data the coarse footprint difference does not predict better performance in the “opposite” condition (unpaired *t*-test of distributions of coarse footprint index in correct vs. incorrect trials was non-significant). This difference, together with our use of brief presentations and masking, further supports that low-level features are insufficient to account for our results. Also, because the correlational structure was tied to shape rather than semantic category (Lin et al., 2014), and because there was no difference in category overlap across stimulus conditions, our results support that the contrastive mechanism was feature-based, not semantically-based.

### Implications for computational models of recognition

An ongoing debate in computational modeling of recognition and generalization learning is how to design the architecture, e.g., whether it is necessary to simulate populations of binary spiking neurons (Masquelier and Thorpe, 2007; Chan et al., 2011; Merolla et al., 2014), or whether convolutional networks are sufficient or even superior. Although convolutional networks outperform spiking networks on datasets like ImageNet, and their performance approximates ideal observers on object categorization and exceeds that of randomly sampled IT neurons (surprising because IT is the last stage of the ventral pathway) (Krizhevsky et al., 2012; Cadieu et al., 2013, 2014; Zeiler and Fergus, 2014), their performance remains far worse than that of humans on real-world vision. In a recent model that approximates IT and ideal observers (Yamins et al., 2014), the approximation to IT is as low as 20%EV for single sites (mean 48.5%EV), and IT split-half data still outperforms the best model on predicting representational dissimilarity for image generalization, object generalization, and category generalization. Our results suggest that part of this gap is due to the much poorer coding capability of soloists vs. choristers and due to the rarity of choristers. We suggest that the comparison (for both convolutional and spiking networks) should be against IT choristers, rather than a random pool of IT neurons. Also, our layer-specific correlation results show that correlation strength increases from input to output layers within a cortical column. Increasing the cell and layer specificity of modeling could in principle favor spiking network models with individual cores that simulate computations within cortical columns, as in Merolla et al. (2014).

Another aspect that may favor spiking network models is the relationship between correlational structure and learning. Whereas learning in convolutional networks occurs via genetic algorithms that guide connection patterns based on overall performance, learning in spiking networks is more local and can in principle be tied to our “pipe cleaner” model and spike timing dependent plasticity (STDP). This difference in approach manifests in convolutional networks as a gradual increase in performance along the hierarchy (Serre et al., 2007), whereas in our data the near-chance performance of soloists hints that performance increase may be staggered along the hierarchy, alternating between low performing input layers and high performing output layers for each cortical “area”. This alternation between low-performing soloists and high-performing choristers may be critical to learning and maintaining an invariant representation (cortically local subspace untangling; DiCarlo et al., 2012). Another aspect that could be modeled is that choristers are rarer in IT (Lin et al., 2014) than in V1 (Chu et al., 2014). We speculate that the increasing rarity of choristers is because of increasing complexity (increasingly high-dimensional feature spaces) along the visual hierarchy, which requires ever larger and more heterogeneous populations of soloists within each column to develop and maintain an invariant low-dimensional (manifold) representation. These emphases on local learning could also benefit from architectures based on multicore networks.

### How might the correlational structure function algorithmically?

How might a homogeneous/heterogeneous spiking network support generalization learning? Our conceptual “pipe cleaner” model (Lin et al., 2014) predicts that the feedforward and feedback inputs to IT may act as tensors, enabling the fine adjustments that may be necessary to build and maintain an invariant representation. The near-orthogonality of the inputs vs. the manifold (Figure 4D) indicates that they are optimally tuned to alter the manifold (i.e., co-alignment with the manifold would be inefficient and could conceivably result in uneven coverage). Such adjustments could occur via STDP, because soloists (mostly in the input layers) that are better tuned to the feedforward (environmental) and feedback (behavioral context) input statistics will spike more quickly, shaping the tuning of the choristers that support the invariant representation. This prediction is consistent with a recent report that found layer-specific temporal sequencing in perirhinal cortex (Takeuchi et al., 2011). Because the invariant representation in IT is based on shape rather than semantic category, invariance training on any category would also improve invariance to other categories that share the same feature, supporting generalization from few examples. We speculate that the combination of heterogeneity (population sparseness) in the input layers and redundancy/smoothness (overlap in tuning) in the output layers may be important for populations of spiking neurons, to achieve sufficient bit resolution from binary spiking neurons operating in high dimensional feature space. This problem of poor bit resolution has been criticized as a fundamental weakness of spiking network models vs. convolutional network models of recognition, and homogeneous/heterogeneous networks may be a key part of the brain’s solution.

### On technical approaches to study efficient coding

Recent technical advances have improved cell-specificity, sampling density, and anatomical co-registration. Our results suggest that, to better understand how the local correlational structure contributes to efficient coding, simultaneous sampling across multiple depths down to at least 1.0–1.2 mm is essential, to map both the inputs and the outputs within a column. Sampling density on the order of 64 neurons/mm^3^ is also critical, to measure correlational structure (not just tuning sharpness) and to detect choristers that are rare in IT. Finally, high temporal resolution is necessary, to link the correlational structure to mechanisms such as STDP and to learning behavior. Currently, dense electrode arrays (e.g., the NeuroNexus Matrix Array) are the only technology that meets these design requirements in terms of deep sampling and sampling individual spikes *in-vivo* in behaving mammals. Unlike other technologies that are still in development, this technology is available today, and its potential for transforming neuroscience remains largely untapped.

## Conflict of interest statement

The authors declare that the research was conducted in the absence of any commercial or financial relationships that could be construed as a potential conflict of interest.
