# Corrigendum: Correlated activity supports efficient cortical processing

**DOI:** 10.3389/fncom.2015.00025

**Published:** 2015-02-24

**Authors:** Chou P. Hung, Ding Cui, Yueh-peng Chen, Chia-pei Lin, Matthew R. Levine

**Affiliations:** ^1^Department of Neuroscience, Georgetown UniversityWashington, DC, USA; ^2^Institute of Neuroscience, National Yang-Ming UniversityTaipei, Taiwan

**Keywords:** object recognition, inferior temporal cortex, macaque, visual search, efficient coding

In the original article, in the second paragraph of the Analyses section, there was a typo in the description of “soloists” that are based on the median 30%ile. They are the 35–65%ile, not the 45–65%ile as previously reported. In addition to the 2nd paragraph of the Analyses section, the typo also appears in 3 other places: Figure 2 legend, 1st paragraph of Results, and in the second paragraph of “Correlated Neurons are Mostly in Output Layers.”

The authors apologize for this error.

## Conflict of interest statement

The authors declare that the research was conducted in the absence of any commercial or financial relationships that could be construed as a potential conflict of interest.

